# The Bone Resorption Inhibitors Odanacatib and Alendronate Affect Post-Osteoclastic Events Differently in Ovariectomized Rabbits

**DOI:** 10.1007/s00223-013-9800-0

**Published:** 2013-10-02

**Authors:** Pia Rosgaard Jensen, Thomas Levin Andersen, Brenda L. Pennypacker, Le T. Duong, Jean-Marie Delaissé

**Affiliations:** 1 Clinical Cell Biology, Institute of Regional Health Research, University of Southern Denmark, Vejle/Lillebaelt Hospital, Vejle, Denmark; 2 Bone Biology Group, Merck Research Laboratories, West Point, PA USA; 3 Clinical Cell Biology, Vejle Hospital, Kabbeltoft 25, 7100 Vejle, Denmark

**Keywords:** Cathepsin K, Bisphosphonate, Osteoclast, Reversal phase, Bone formation, Coupling of bone resorption and formation

## Abstract

Odanacatib (ODN) is a bone resorption inhibitor which differs from standard antiresorptives by its ability to reduce bone resorption without decreasing bone formation. What is the reason for this difference? In contrast with other antiresorptives, such as alendronate (ALN), ODN targets only the very last step of the resorption process. We hypothesize that ODN may therefore modify the remodeling events immediately following osteoclastic resorption. These events belong to the reversal phase and include recruitment of osteoblasts, which is critical for connecting bone resorption to formation. We performed a histomorphometric study of trabecular remodeling in vertebrae of estrogen-deficient rabbits treated or not with ODN or ALN, a model where ODN, but not ALN, was previously shown to preserve bone formation. In line with our hypothesis, we found that ODN treatment compared to ALN results in a shorter reversal phase, faster initiation of osteoid deposition on the eroded surfaces, and higher osteoblast recruitment. The latter is reflected by higher densities of mature bone forming osteoblasts and an increased subpopulation of cuboidal osteoblasts. Furthermore, we found an increase in the interface between osteoclasts and surrounding osteoblast-lineage cells. This increase is expected to favor the osteoclast–osteoblast interactions required for bone formation. Regarding bone resorption itself, we show that ODN, but not ALN, treatment results in shallower resorption lacunae, a geometry favoring bone stiffness. We conclude that, compared to standard antiresorptives, ODN shows distinctive effects on resorption geometry and on reversal phase activities which positively affect osteoblast recruitment and may therefore favor bone formation.

## Introduction

Bone diseases, such as osteoporosis and most cancer metastases to bone, are characterized by loss of bone mass and increased fracture risk. This is largely due to excessive bone resorption compared to bone formation during bone remodeling. These skeletal diseases have triggered the development of a series of inhibitors of osteoclasts, the cells responsible for bone resorption. These inhibitors comprise estrogen, selective estrogen receptor modulators (SERMs), calcitonin, bisphosphonates such as alendronate (ALN), as well as the newly developed denosumab [1]. Although these antiresorptive drugs are demonstrated to improve bone mineral density and to reduce fracture risks in patients, they are also known to reduce bone formation as a consequence of the coupling between resorption and formation during bone remodeling. Recent efforts have aimed at finding alternative treatments based on stimulation of osteoblasts, the bone forming cells. These have brought intermittent parathyroid hormone (PTH) therapy onto the market and anti-sclerostin into clinical trials [2]. However, despite their clinical efficiency, the bone formation induced by these anabolic agents is not strictly linked to the sites of bone resorption, and they may not reestablish the site-specific bone resorption–bone formation balance that is lost in pathological bone remodeling [3, 4].

Odanacatib (ODN) is a newly developed inhibitor of osteoclastic bone resorption presently in a clinical phase III trial. It is unique compared with the standard antiresorptives because of its ability to inhibit bone resorption while keeping bone formation generally unaffected or even stimulated at specific bone sites, as supported by observations in clinical and preclinical studies with ovariectomized (OVX) monkeys and rabbits [5–7]. ODN is therefore considered to represent a new generation of bone resorption inhibitors that may bring alternative benefits to osteoporotic patients compared to the current standard of care. The mechanism whereby ODN inhibits bone resorption is well understood [8, 9]; however, how ODN preserves bone formation requires further investigations.

Osteoclasts remove bone by secreting protons and cathepsin K, a potent collagen-degrading proteinase [10]. The protons solubilize the mineral, thereby denuding the collagen fibers, which make up 90 % of the organic bone matrix. Once denuded, collagen becomes available to cathepsin K. ODN is a specific inhibitor of cathepsin K and inhibits thus the resorptive activity of the osteoclast by inhibiting collagen degradation [8, 9]. This means that ODN inhibits the very last step of the resorption process, while other anti-osteoclastic drugs, like bisphosphonates and anti-RANKL antibody, act more upstream. We speculated that by uniquely inhibiting this very last step of the resorption process, ODN could also uniquely modify the signals left in the resorption lacunae vacated by the osteoclasts and therefore also modify the cellular events which follow immediately after osteoclastic resorption, such as osteoblast recruitment. These events are obviously important in connecting bone resorption and formation. We thus hypothesized that ODN might affect bone formation—in contrast to classical anti-osteoclastic drugs—by acting on this connecting step, known as the “reversal phase” [11]. Interestingly, inhibition of cathepsin K by a broad-spectrum cysteine proteinase inhibitor induced unusual features at the reversal phase in a mouse model [12], and similar observations were made in a rabbit model [13] and in pycnodysostosis, a disease caused by a mutation in the gene encoding cathepsin K [12]. It is worth noting that a drug affecting the reversal phase may be of special relevance to osteoporosis because this disease is precisely characterized by a lack of coordination between resorption and formation, and prolonged or arrested reversal phase has been reported in estrogen deficiency– and age-related bone loss [11, 14]. Although these reports point to a failure at the level of the reversal phase, a phase linking resorption and formation has remained largely undercharacterized [11, 14].

The present study aimed at identifying what differentiates ODN from classical anti-osteoclastic drugs, such as ALN, in the OVX rabbit model [7]. We histomorphometrically compared their effects on the target cell, the osteoclast, and on the post–osteoclastic events of the reversal phase. It is a follow-up of a previous histomorphometric study performed on OVX rabbits, where ODN was shown to differ from ALN in its ability to preserve bone formation [7]. The recent report on this earlier study strictly concerned the extent of mineralization surfaces and bone formation rates and did not show data either on the resorption or on the reversal phase, which we here hypothesize to determine the subsequent bone formation levels.

## Materials and Methods

### Rabbit Bone Samples

The present study is a follow-up of the recent study reported by Pennypacker et al. [7]. In brief, 6-month-old female rabbits were randomized into four groups: (1) sham-operated (*n* = 11), (2) OVX and treated with vehicle (*n* = 12), (3) OVX and treated subcutaneously with ALN (300 μg/kg twice/week, *n* = 11), and (4) OVX and treated orally with ODN given in a control diet (Teklad global high fiber 2031C; Harlan, Indianapolis, IN) at a concentration of 0.0016 % (w/w) corresponding to a steady-state exposure of AUC = 4 μM/24 h of ODN (*n* = 12). Since the plasma protein binding is lower in the rabbit (87.3 %) than in humans (97.5 %), the free fraction of ODN exposure in this study was 0.51 μM/24 h, or approximately threefold of the human daily free fraction calculated from the 50-mg once-weekly clinical dose (6–7 μM/24 h). Treatment of the rabbits was started on the third day after ovariectomy and continued for 27 weeks, after which the animals were killed. For histomorphometry, lumbar vertebra L4 was removed, immersed in 70 % ethanol, and embedded undecalcified in 80 % methyl methacrylate–20 % dibutyl phthalate. For immunohistochemistry, bone specimens (L2) were obtained from a new study including three rabbits in each of the four groups. The four groups were treated as described above except for the ODN group, which was treated orally in the control diet at a concentration of 0.0043 % (w/w) corresponding to a steady-state exposure of AUC = 7.5 μM/24 h of ODN, and the ALN group, which was treated once weekly at a dose of 300 μg/kg. Bone specimens were fixed in 4 % formaldehyde, decalcified in 0.2 M EDTA-Na_2_ containing 0.05 % formaldehyde, and embedded in paraffin.

### Histochemistry and Immunohistochemistry

Paraffin sections (3.5 μm thick) from L2 were processed for toluidine blue (1 %), tartrate-resistant acid phosphatase (TRAP) activity, and immunohistochemical stains. Toluidine blue staining was performed as previously described [6]. TRAP activity staining was done as described by van de Wijngaert and Burger [15], sections were counterstained with Mayer’s hematoxylin and mounted. For immunohistochemistry, the procedure included deparaffinization and rehydration of the sections, followed by epitope retrieval in Tris–EDTA buffer (pH 9.0) overnight at 60 °C. Immunostainings for cathepsin K were performed using a polyclonal goat IgG antibody (sc-6507; Santa Cruz Biotechnology, Santa Cruz, CA, USA) as primary antibody and a mouse anti-goat IgG (Jackson ImmunoResearch Laboratories, West Grove, PA, USA) as secondary antibody. The mouse anti-goat IgGs were detected with a polymeric alkaline phosphatase–conjugated system (PowerVision; ImmunoVision Technologies, Hillsborough, CA, USA) and visualized by liquid permanent red (DakoDenmark, Glostrup, Denmark). Sections were counterstained with Mayer’s hematoxylin and mounted.

### Histomorphometry and Electron Microscopy

Histomorphometric parameters were assessed in the cancellous bone of Masson-Goldner trichrome–stained sections (6 μm) prepared from the plastic-embedded L4 as described [7]. The parameters included the trabecular bone volume per tissue volume (BV/TV), trabecular thickness (Tb.Th), trabecular number (Tb.N), trabecular spacing (Tb.Sp), as well as the fraction of trabecular bone surface covered by eroded (ES/BS), osteoclast (Oc.S/BS), reversal (Rv.S/BS), osteoid (OS/BS), and osteoblast surface (Ob.S/BS). Reversal surfaces were defined as eroded surfaces (identified through broken lamellae) without osteoclasts. For every single hit on reversal perimeters the presence of osteoid in the vicinity was recorded. Vicinity was defined as being within the same 2D remodeling unit as the reversal surface itself. Furthermore, discrimination was made between cuboidal (C.Ob.S) and flat osteoblasts based on the cell morphology. Cuboidal osteoblasts were defined as cells with a cuboidal or plumb shape lining osteoid, and flat osteoblasts were defined as cells with a flat nucleus and thin cytoplasm lining osteoid. The trabecular bone volume, trabecular thickness, trabecular number, and trabecular spacing were measured at a total magnification of 100× using the Osteomeasure Analysis System (OsteoMetrics, Decatur, GA, USA), whereas all other histomorphometric analyses were done in a systematic random way at a total magnification of 200× using a light microscope (DM2500; Leica, Herlev, Denmark) equipped with an eyepiece containing a Merz graticule with parallel curved lines [16].

Osteoclasts were characterized in more detail in Masson-Goldner trichrome– and toluidine blue–stained sections. The total number of osteoclast profiles in the cancellous area of the vertebral body was assessed in Masson-Goldner trichrome–stained sections from each rabbit. Discrimination was made between profiles of osteoclasts attached to the bone matrix and profiles of osteoclasts detached from the bone matrix. Profiles of detached osteoclasts were categorized as either osteoclast profiles separated from the bone surface by a layer of mononucleated cells or osteoclast profiles found farther away from the bone surface, i.e., more than one cell layer from the bone surface or above bone forming osteoblasts. Regarding the attached osteoclast profiles, discrimination was made between profiles found in resorption cavities more than two lamellae deep and profiles found on noneroded or shallow cavities, i.e., less than or equal to a depth of two lamellae. This analysis was done at a total magnification of 200× using a light microscope (Leica DMRXAZ), where broken lamellae were visualized by polarized light. The presence of demineralized collagen at the osteoclast–bone interface and the presence of intracellular vesicles in osteoclasts were analyzed in Masson-Goldner trichrome– and toluidine blue–stained sections, respectively, at a total magnification of 400×.

Cell densities of reversal cells and osteoblasts were measured at a total magnification of 400× using the Osteomeasure Analysis System (OsteoMetrics). Here, all cell profiles connected to the bone surface were counted regardless of the presence of a visible nucleus. Samples for electron microscopic analysis were prepared as previous described [17] and analyzed with a transmission electron microscope (TEM 208; Philips, Eindhoven, The Netherlands) operated at 80 kV.

### Statistical Analysis

Data for each group were tested for normality using the D’Agostino-Pearson omnibus test. When the data allowed for parametric statistics, the unpaired *t*-test was used, applying the Welch correction in the presence of unequal variances. When nonparametric statistics were needed, the Mann–Whitney test was used. The correlation between two variables was analyzed using the Spearman rank correlation test. Best-fitted lines were made by linear regression including a comparison of the slopes of the regression lines. *p* < 0.05 was considered statistically significant. All statistical analyses were performed with GraphPad Prism 5 (GraphPad Software, La Jolla, CA,USA).

## Results

### Effects of OVX and ODN or ALN on Structural Indices of Trabecular Bone

In agreement with other reports [18, 19], OVX significantly reduced bone volume and trabecular numbers, significantly increased trabecular space, and did not affect trabecular thickness, thus supporting estrogen deficiency–induced bone loss (Table 1). According to the present measurements, ALN or ODN treatment of OVX rabbits induced thicker trabeculae but did not prevent either the reduction in trabecular number or the increase in trabecular space, as was also reported in OVX monkeys treated with ODN [20].

**Table 1:** Effects of OVX, ALN and ODN on structural indices of trabecular bone

Group	*n*	BV/TV (%)	Tb.Th (μm)	Tb.N (#/mm)	Tb.Sp (μm)
Sham	11	15 ± 3	102 ± 14	1.7 (1.3, 2.2)	456 (342, 628)
OVX + veh	12	11 ± 2**	101 ± 14	1.2 (1, 1.5)*	726 (576, 912)*
OVX + ALN	11	15 ± 5^††^	124 ± 26* ^†^	1.3 (1.1, 1.6)*	688 (511, 783)*
OVX + ODN	12	12 ± 3	121 ± 16** ^††^	1.2 (0.9, 1.5)	735 (554, 1049)

BV/TV and Tb.Th are expressed as mean values ± SD and Tb.N and Tb.Sp as median values, where the first number in parentheses is the first quartile and the second number is the third quartile

* p < 0.05 against sham, ** p < 0.01 against sham, †* p* < 0.05 against OVX + veh, ††* p* < 0.01 against OVX + veh

*OVX* ovariectomy, *ALN* alendronate, *ODN* odanacatib, *BV/TV* bone volume/total volume, *Tb.Th* trabecular thickness, *Tb.N* trabecular number, *Tb.Sp* trabecular spacing

### Effects of ODN and ALN on Osteoclasts and Bone Resorption in OVX Rabbits

**Fig. 1:**
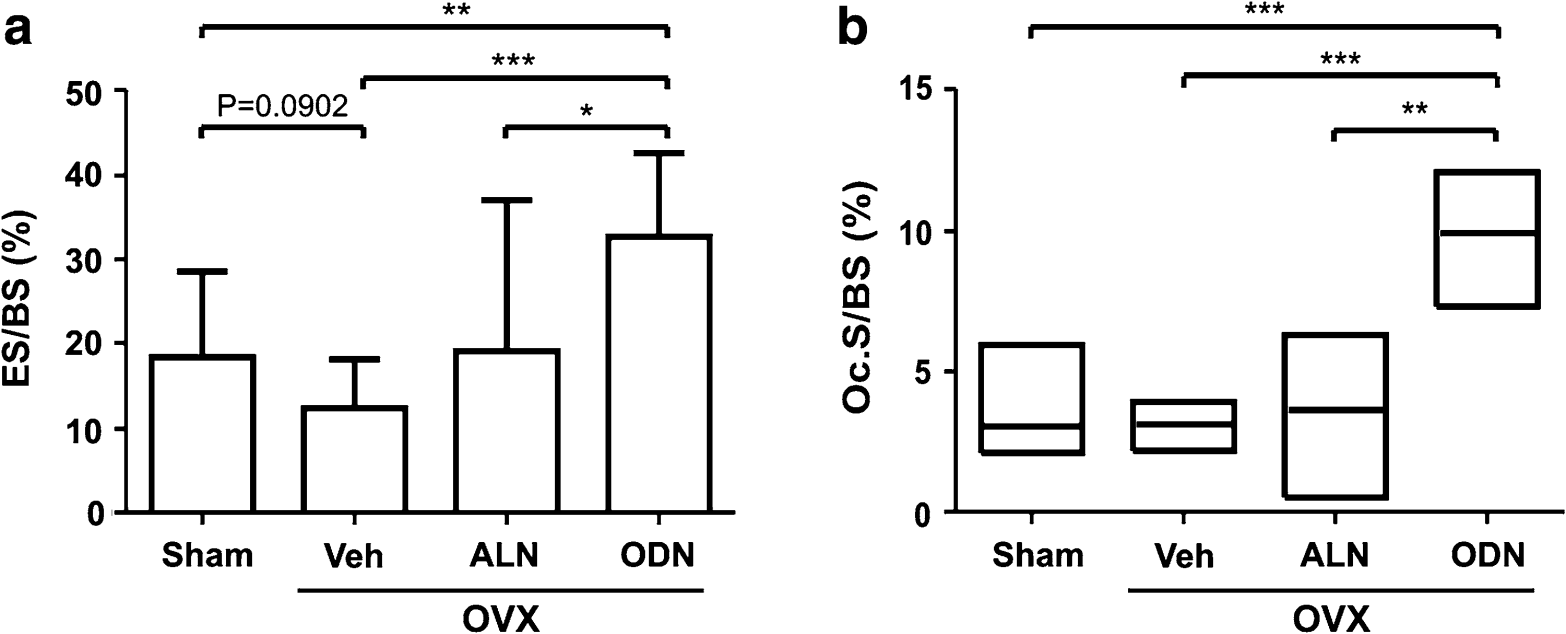
Effect of odanacatib (*ODN*) and alendronate (*ALN*) treatment on bone resorption parameters in ovariectomized (*OVX*) rabbits. The extent of eroded surface (*ES/BS*) and osteoclast surface (*Oc.S/BS*) was assessed in the vertebral trabecular bone of sham and OVX rabbits treated with vehicle (*Veh*), ALN, or ODN. The results are shown as means with SD **a** or as medians with upper and lower quartiles **b**. Differences between the groups were analyzed by the Mann–Whitney test **b** or the *t*-test **a**, applying Welch correction in the presence of unequal group variances. *p < 0.05, **p < 0.01, ***p < 0.001

Since both ODN and ALN target the osteoclast, we performed a systematic analysis of the extent of bone surface covered by eroded surface and by osteoclasts. Vehicle-treated OVX rabbits (OVX + Veh) did not demonstrate any significant changes in the extent of eroded surface or osteoclast surface compared to the sham group (Fig. 1), which is consistent with other long-term OVX studies [19]. Treatment of OVX rabbits with ODN resulted in a twofold increase in the extent of eroded surface and a threefold increase in osteoclast surface, whereas ALN did not induce any significant changes in these parameters. These observations are consistent with earlier reports on ODN effects in monkeys [6, 21] and on ALN effects in multiple species [22–24].

**Fig. 2:**
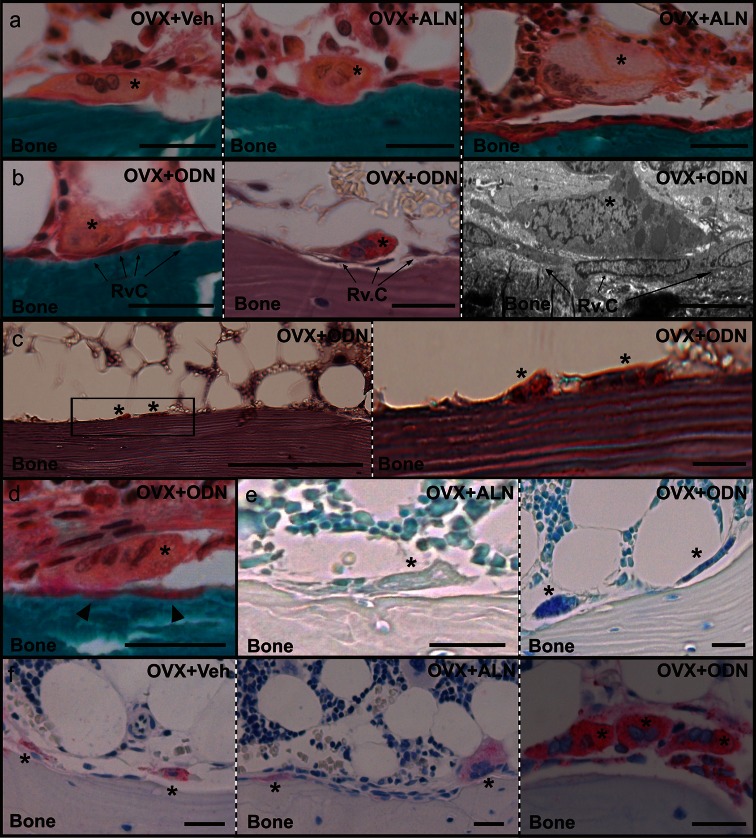
Histological appearance of osteoclasts in odanacatib (*ODN*)– and alendronate (*ALN*)–treated ovariectomized (*OVX*) rabbits. **a** Masson-Goldner trichrome staining shows the appearance of osteoclast profiles (*asterisks*) attached to the vertebral trabecular bone surface from a vehicle-treated (*left panel*) and an ALN-treated (*middle panel*) OVX rabbit. Osteoclast profiles frequently appeared away from the bone surface in ALN-treated OVX rabbits as shown by the presence of a giant osteoclast (*asterisk*) above bone forming osteoblasts (*right panel*). **b** Examples of osteoclast profiles (*asterisks*) detached from the bone surface by mononucleated reversal cells (*Rv.C*, *arrows*) in an ODN-treated OVX rabbit as they appear using Masson-Goldner trichrome staining (*left panel*), TRAP activity staining (*middle panel*), and electron microscopy (*right panel*). **c** TRAP-positive osteoclast profiles (*asterisks*) attached to a bone surface without visible broken lamellae. The *framed area* in the *left panel* is enlarged in the *right panel*. **d** Example of an osteoclast profile (*asterisk*) with demineralized collagen below (*arrowheads*), visualized by Masson-Goldner trichrome staining. **e** Toluidine blue staining showing the absence and presence of intracellular vesicles in osteoclast profiles (*asterisks*) in the vertebral trabecular bone from an ALN-treated (*left panel*) and an ODN-treated (*right panel*) OVX rabbit. **f** Immunohistochemical staining showing elevated intracellular levels of cathepsin K in trabecular osteoclasts (*asterisks*) of ODN-treated OVX rabbits (*right panel*) compared to ALN-treated (*middle panel*) and vehicle-treated (*left panel*) OVX rabbits. Scale bar = 50 μm, except for **b**, *right panel* = 10 μm and **c**, *left panel* = 200 μm

**Fig. 3:**
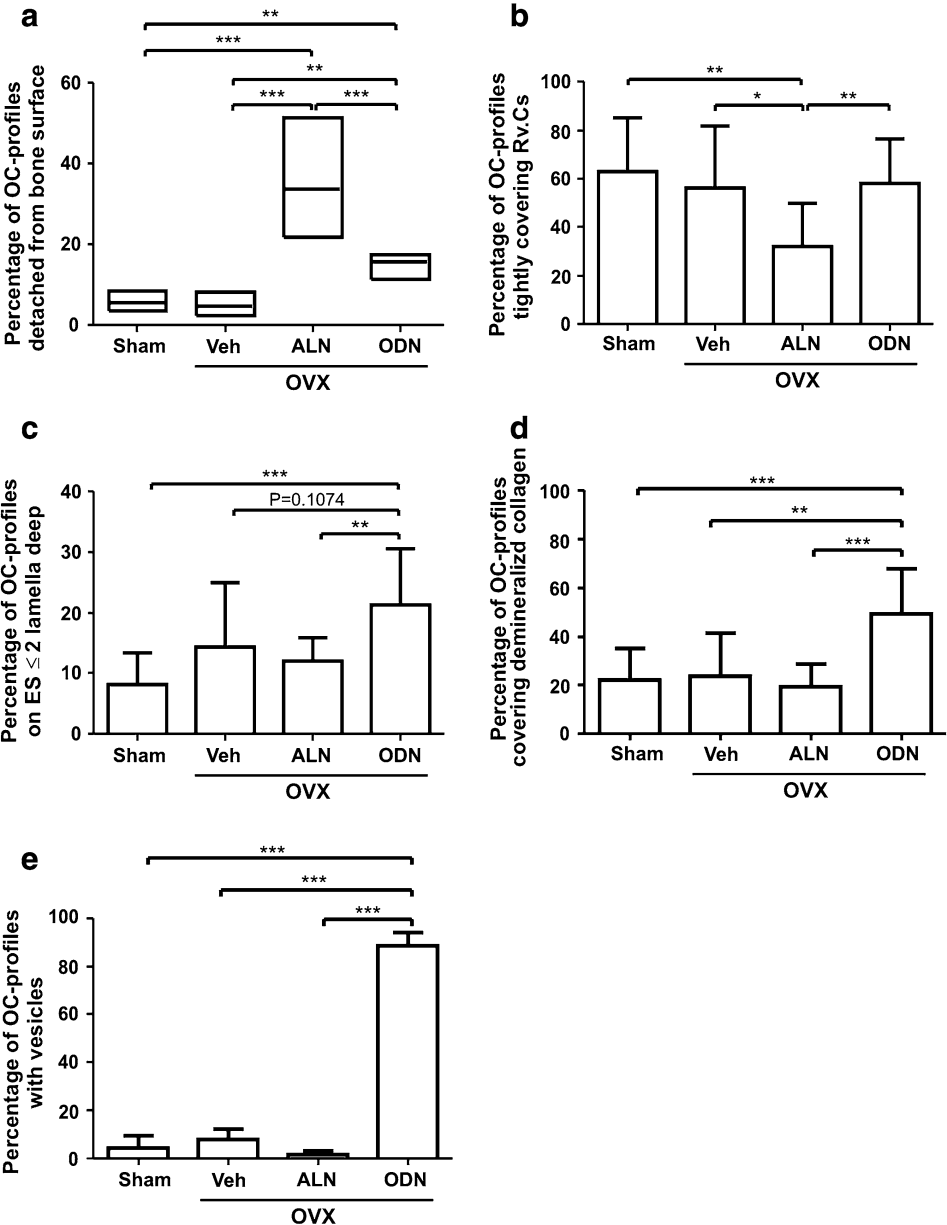
Effects of odanacatib (*ODN*) and alendronate (*ALN*) treatment on osteoclast features. **a** The extent of osteoclast profiles detached from the bone surface was evaluated in the vertebral trabecular bone of sham and ovariectomized (*OVX*) rabbits treated with vehicle (*Veh*), ALN, or ODN. Data are expressed as the percentage of detached osteoclast profiles per total number of osteoclast profiles. **b** Among the osteoclast profiles detached from the bone surface, some covered tightly reversal cells (*Rv.Cs*). The fraction of this population of osteoclasts is expressed as percentage of the total detached osteoclasts. **c** Percentage of osteoclast profiles attached onto bone surfaces with an erosion depth of ≤2 lamellae. **d** Percentage of osteoclast profiles with demineralized collagen below. **e** Percentage of osteoclast profiles with intracellular vesicles. Results are shown as medians with upper and lower quartiles **a** or as means with SD **b–e**. Rabbits with fewer than 20 osteoclast profiles were excluded from the analyses. Differences between groups were analyzed by the Mann–Whitney test **a** or the *t*-test **b–e**, applying the Welch correction in the presence of unequal group variances. *p < 0.05, **p < 0.01, ***p < 0.001

Because osteoclasts responded differently to ODN and ALN, a more thorough examination of the treatment-related effects on osteoclasts was undertaken. Osteoclast profiles were generally found in contact with the bone surface in the OVX + Veh group (Fig. 2a). Although this was also the case for the majority of the osteoclast profiles in the ALN- and ODN-treated OVX rabbits, a substantial proportion of the profiles appeared detached from the bone surface (Fig. 2a, b). Upon quantification, the proportion of detached osteoclast profiles proved to be increased sixfold in ALN- and threefold in ODN-treated OVX rabbits compared to vehicle-treated OVX rabbits (Fig. 3a). The proportion of detached osteoclast profiles was thus two times higher in the group receiving ALN than in the group receiving ODN (Fig. 3a). Further analysis on the detached osteoclast profiles revealed that more than half of these profiles in sham, vehicle-, and ODN-treated OVX rabbits were separated from the bone surface by a single layer of mononucleated cells, i.e., reversal cells, which were found directly interposed between the osteoclast itself and the bottom of the resorption cavity (Figs. 2b, 3b). In contrast, the fraction of osteoclast profiles showing this type of contact with reversal cells was two times lower in the ALN versus ODN treatment. Instead, in the presence of ALN, the detached osteoclast profiles were more frequently found at ≥2 cell layers away from the bone surface or even above bone forming osteoblasts (Figs. 2a, 3b), thus suggesting that ALN treatment changes the adhesion and function of the osteoclasts more dramatically than ODN.

Another unique effect of ODN concerns the bone in contact with osteoclast profiles. In ODN-treated OVX rabbits, osteoclasts appeared about twice as frequently on noneroded surfaces or shallow cavities (≤2 lamellae deep) compared to the situation in the other groups, including ALN treatment (Figs. 2c, 3c). Hence, even though ODN treatment leads to an increased extent of osteoclast and eroded surfaces, erosion is not deep and the total amount of resorbed bone matrix should not necessarily be as significant as suggested by the extent of eroded surfaces.

Staining of undegraded, demineralized collagen frequently appeared at the osteoclast–bone interface in ODN-treated OVX rabbits (Figs. 2d, 3d), as was previously demonstrated in mouse and rabbit bones treated with a broad-spectrum cysteine proteinase inhibitor [12, 13]. Furthermore, ODN treatment induced an accumulation of toluidine blue–stained intracellular vesicles in osteoclasts (Figs. 2e, 3e), as reported earlier in ODN-treated monkeys and interpreted as accumulation of collagen fragments [6]. No stained vesicles were observed in sham, vehicle-, or ALN-treated OVX rabbits (Fig. 3e). These observations thus indicate compromised extracellular and intracellular degradation of demineralized collagen in the presence of ODN, in line with earlier reports on the effect of cathepsin K inhibition [8, 12, 25].

Interestingly, the immunoreactivity of cathepsin K appeared to be increased in ODN-treated OVX rabbits compared to sham, vehicle-, and ALN-treated OVX rabbits (Fig. 2f), which suggests that increased expression/intracellular accumulation of cathepsin K is induced by ODN treatment. This observation is consistent with earlier observations obtained in various experimental systems [8, 26, 27].

### Effects of ODN and ALN Treatment on the Reversal Phase

**Fig. 4:**
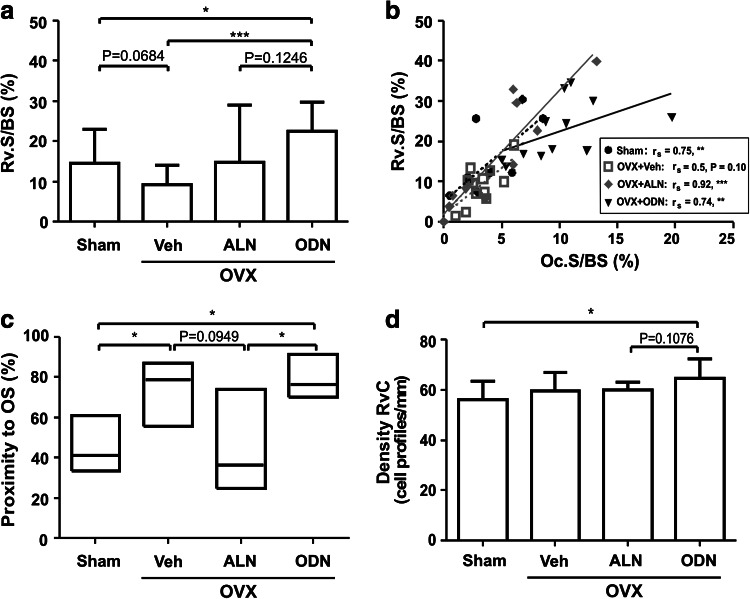
Effect of odanacatib (*ODN*) and alendronate (*ALN*) treatment on the reversal phase. **a** The extent of reversal surface (*Rv.S/BS*) was assessed in the trabecular bone of a vertebra of sham and OVX rabbits treated with vehicle (*Veh*), ALN, or ODN. **b** The Rv.S shown in **a** was plotted against the Oc.S/BS shown in Fig. 1 in order to evaluate the relationship between osteoclast and reversal phase prevalence for each rabbit. Each dot represents the measurement obtained in a bone specimen isolated from one rabbit (sham: *black circles, black dotted line*; OVX + Veh *gray open squares, gray dotted line*; OVX + ALN *gray diamonds, gray line*; OVX + ODN *black triangles, black line*). **c** The proximity of the reversal surfaces to formative surfaces is shown for each group as the percentage of Rv.S with OS in the vicinity as defined in “Materials and Methods.” **d** The density of mononucleated cells on the reversal surfaces, i.e., reversal cells (*Rv.C*), is shown for each of the four groups of rabbits in cell profiles per millimeter. Results are shown as means with SD **a**, **d** or medians with upper and lower quartiles **c**. The Mann–Whitney **c** or the *t*-test **a**, **d** was used to analyze whether the parameters were significantly different in the four groups, and correlations between the extent of Oc.S/BS and Rv.S/BS **b** were analyzed using the Spearman rank correlation test, *p < 0.05, **p < 0.01, ***p < 0.001. The lines in **b** were made by linear regressions. Comparison of the slopes of the regression lines for sham, OVX + Veh, and OVX + ALN revealed no differences. Therefore, the slope of the best-fitted regression line for the pooled groups (line not shown, Spearman rank correlation for pooled groups *r*_s_ = 0.80, *p* < 0.0001) was compared with the slope of the regression line for OVX + ODN and proved to be significantly different (*p* = 0.0012)

The reversal surfaces are defined as eroded surfaces vacated by osteoclasts [11]. They are the surfaces that physically connect bone resorption to formation, meaning that they may play a critical role in the initiation of bone formation during bone remodeling. Because ODN and ALN affect osteoclasts differently, it is important to analyze whether this difference has an impact on the reversal phase. We first analyzed the extent of reversal surfaces in the four groups of rabbits (Fig. 4a). Ovariectomy of the rabbits (OVX + Veh) had no significant effect on the extent of reversal surfaces compared to sham. Compared to OVX + Veh rabbits, ODN treatment increased the extent of reversal surface more than twice, whereas ALN treatment induced a widespread distribution of the values, with no significant effect being found (Fig. 4a). Furthermore, when relating the extent of reversal surface to the extent of osteoclast surface measured in each biopsy, a clear positive correlation was observed in all groups, meaning that the more bone surface is covered by osteoclasts, the larger is the extent of reversal surfaces (Fig. 4b). Interestingly, however, as indicated by the slope of the regression lines, the increases in reversal surface were significantly smaller in the ODN group compared with all of the other groups, suggesting that osteoid deposition starts sooner. In line with this, reversal surfaces were more than twice as frequently close to osteoid surfaces in the presence of ODN than in the presence of ALN (Fig. 4c). Furthermore, compared to all of the other groups, cell densities on the reversal surfaces tended to increase in ODN-treated OVX rabbits, which was the only group showing a significantly increased reversal cell density compared with sham (Fig. 4d). Altogether these observations indicated that ODN and ALN affected the reversal phase differently and thereby also the connection of resorption and formation. Thus, compared to ALN, ODN appeared to allow higher bone surface cell densities and a faster initiation of the bone formation step during bone remodeling.

### Effects of ODN and ALN Treatment on Bone Formation Parameters

**Fig. 5:**
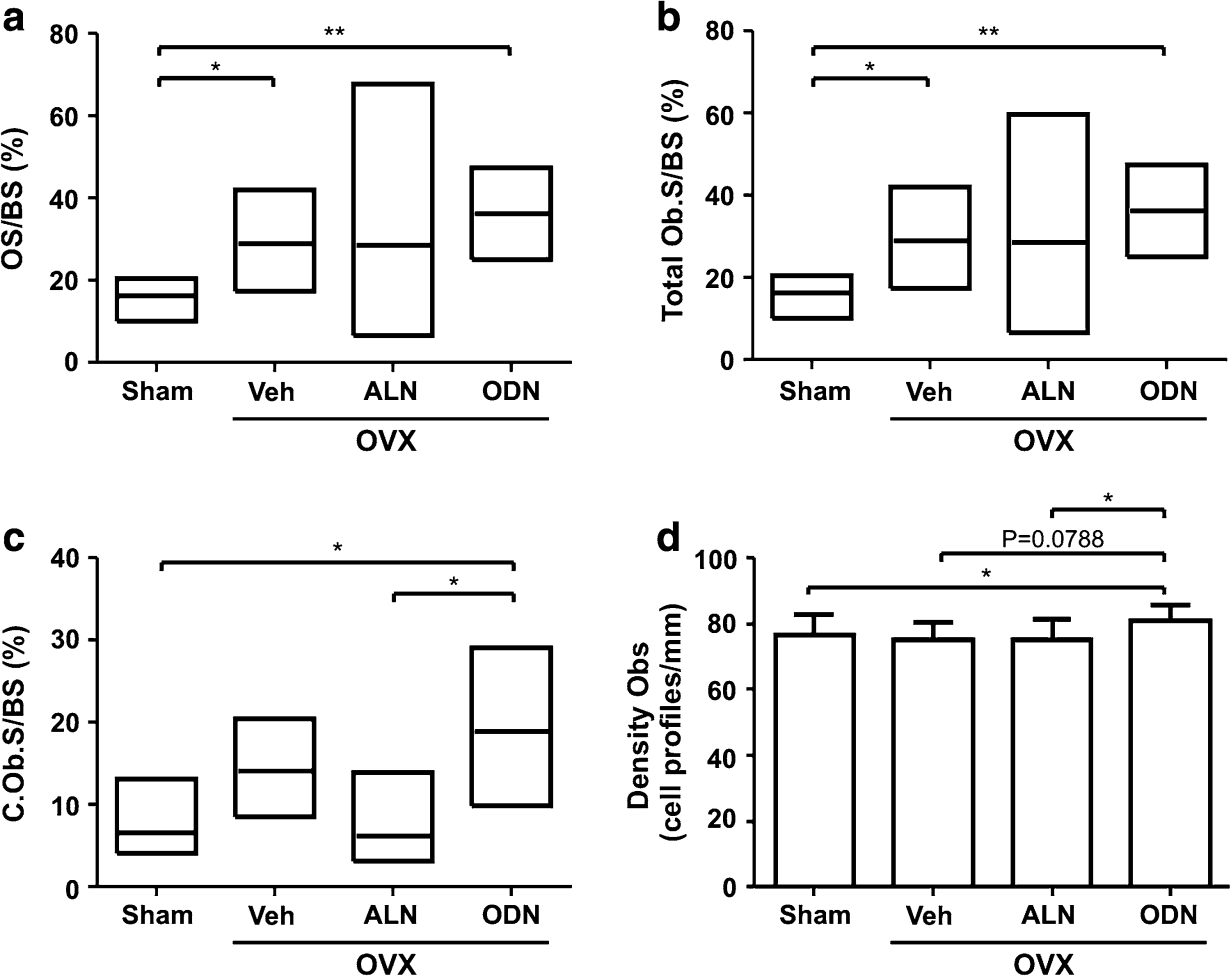
Effect of odanacatib (*ODN*) and alendronate (*ALN*) treatment on bone formation parameters. The extent of osteoid surface (*OS/BS*) **a**, total osteoblast surface (*Ob.S/BS*) **b**, and cuboidal osteoblast surface (*C.Ob.S/BS*) **c** was evaluated in a vertebra from the sham and OVX rabbits treated with vehicle (*Veh*), ALN, or ODN. **d** The density of osteoblasts (cell profiles per millimeter) is shown for each of the four groups. Results are shown as medians with upper and lower quartiles **a–c** or means with SD **d**. The Mann–Whitney **a–c** or the *t*-test **d** was used to analyze whether the parameters were significantly different in the four groups. *p < 0.05, **p < 0.01

In the previous report on the comparative effects of ODN and ALN in OVX rabbits [7], mineralizing surfaces and bone formation rates were shown to increase in response to OVX and to be higher upon treatment of the OVX rabbits with ODN compared with ALN. Here, we analyzed additional bone formation parameters. Figure 5(a, b) shows that the extent of osteoid and osteoblast surfaces also increased in response to OVX but that they were not changed upon further treatment with ODN or ALN. In this respect, one should note that the measurements in the ALN group were widely distributed, with values ranging from approximately 2–87 %, thus revealing variable effects of ALN on the extent of osteoid and osteoblast surfaces.

Interestingly, however, a more detailed analysis demonstrated clearly distinct effects of ODN and ALN on osteoblasts. Compared with ALN, ODN led to a threefold higher prevalence of cuboidal osteoblasts (Fig. 5c), a morphology reflecting a higher cell density. In line with this observation, ODN also led to significantly higher osteoblast densities compared with both ALN-treated and sham rabbits (Fig. 5d). Thus, overall, the higher prevalence of cuboidal osteoblasts and the higher osteoblast density may contribute to the increased mineralizing surfaces and bone formation rates observed upon ODN treatment compared with ALN treatment [7].

## Discussion

It is well known that ODN and ALN have in common to be inhibitors of osteoclastic bone resorption, but that ODN differs from ALN and other traditional antiresorptives by its ability to preserve bone formation. The present study emphasizes a series of additional distinctive properties of ODN. These relate to bone resorption itself, the reversal phase, and osteoblast recruitment. Interestingly, the effects of ODN on the latter two shed insight into how ODN may favor bone formation.

Regarding resorption, our data show that treatment with ODN clearly changes the geometry of the resorption cavities compared to controls and a classical antiresorptive, ALN. Upon ODN treatment, a larger surface is eroded but erosion is less deep. The latter characteristic is in accordance with the repeated demonstration of shallower excavations in the pit assay in response to lowered levels of cathepsin K activity [28–31]. It is of interest that ODN thereby mimics the effect of estrogen, which reduces both cathepsin K expression [32, 33] and the depth of the pits [30]. As discussed by the latter authors, a shallower geometry may help in preventing perforations of trabeculae; and it was also shown to favor bone stiffness [34]. Thus, a unique property of ODN is to allow bone remodeling while simply reducing its rate and changing the geometry of the package of matrix, which is renewed with less risk of fragilization. This effect on geometry is probably a result of ODN slowing down collagen degradation without directly affecting demineralization. The rate of collagen degradation relative to the rate of demineralization is a major determinant of the shape of the resorption lacunae, as discussed by Soe et al. [35]. It should be noted that others also reported that ALN has no effect on erosion depth [23].

The reversal phase has been proposed to prepare the eroded bone surfaces for bone formation [12], and reversal phase arrest prevents subsequent bone formation [11, 12, 14]. The reversal phase is thus a key for successful remodeling. As explained below, ODN shows unique effects on the reversal phase compared to ALN and control: (1) it shortens it, thus shortening the duration between the end of resorption and the initiation of bone deposition, and (2) it favors osteoblast recruitment.

In earlier studies, the duration of the reversal phase was estimated by relating the extent of the reversal surface to the extent of the osteoclast surface, and it was shown that reversal surfaces relative to osteoclast surfaces were smaller in situations where resorption is known to be ideally coupled with formation, like in primary hyperparathyroidism [11, 14]. In contrast, it was shown to be larger in situations of less efficient coupling, like in osteoporosis [11, 14]. In the present study, the ODN-induced shortening of the reversal surface relative to the osteoclast surface thus points to an efficient coupling and faster initiation of bone formation in the presence of ODN compared to all other conditions investigated here. This interpretation is supported by the fact that bone deposition is more frequently seen in the neighborhood of reversal surfaces upon ODN treatment than in all the other conditions.

A major event occurring during the reversal phase is recruitment of osteoblasts to the bone surface. It should be noted that the result of this process is not well reflected by the extent of osteoblast surface since osteoblast-lineage cells may spread to a variable extent over the bone surface, where they may adopt either a flat or a cuboidal morphology. The result of osteoblast recruitment is thus better appreciated through cell density. Here, we show that ODN treatment favors higher osteoblast densities and more cuboidal osteoblasts compared to ALN treatment. It makes sense to believe that this higher cell density would result in higher bone formation rates and mineralizing surfaces, as previously reported by Pennypacker et al. [7]. Interestingly, cell densities appeared already higher at the level of the reversal phase, which is in accordance with the view that reversal cells may differentiate into mature osteoblasts.

What is the mechanism whereby ODN treatment affects the reversal phase? Here, two issues should especially be emphasized. First, the signals left by the osteoclast in the resorption lacuna are probably also the signals seen by the cells colonizing these lacunae after the departure of the osteoclast. Since ODN acts on the very last step of the resorption process, ODN treatment is likely to affect these signals. Obvious changes due to the presence of ODN are less efficient degradation of organic matrix molecules, thus resulting in the accumulation of demineralized collagen; of other bone matrix molecules [25]; as well as of matrix-associated growth factors [36]. Collagen and growth factors, like transforming growth factor-β, have been shown to be chemoattractants for osteoblast-lineage cells [37, 38]. Furthermore, electron microscopic studies of mouse and rabbit bone cultures performed in the presence of cathepsin K inhibitors showed numerous osteoblast-lineage cells in close contact with osteoclasts and enwrapping the demineralized collagen fibers that the osteoclasts themselves were unable to degrade [12, 13]. The same osteoblast-lineage cells were also shown to deposit cement lines and collagen fibers in the vacated pits [12, 39]. Here, we have extended these studies in several ways: we used a selective cathepsin K inhibitor, ODN; we quantified the interactions of the osteoclasts with the periosteoclastic cells; and we showed that ODN favors these interactions compared to ALN. Taking these observations together, a likely scenario contributing to increased osteoblast recruitment is that collagen leftovers and undegraded growth factors stimulate the recruitment and the activity of the cells colonizing the vacated resorption lacunae, which are the cells that subsequently differentiate into mature bone forming osteoblasts.

In relation with the mechanism whereby ODN affects the reversal phase and bone formation, one should also emphasize the impressive increase in osteoclast surface induced by ODN. Several other situations of increased osteoclast surface have been reported, which include impairment of c-Src and chloride channel 7 (ClC-7) activity [40, 41]. Interestingly, all these situations have been associated with increased bone formation [41, 42]. This effect is poorly understood and has led to the proposal that osteoclastic anabolic factors might be involved [43]. One may also note that all these situations, including the ODN-induced increase in osteoclast prevalence, lead to an increase in the interface between osteoclasts and all cells surrounding the osteoclasts. Among the latter cells are not only reversal cells, as discussed above, but also (1) bone remodeling compartment canopy cells, which are osteoblast-lineage cells and likely contribute to the formation of mature bone forming osteoblasts [16, 44], and (2) endothelial cells, which appear also to be involved in bone formation during remodeling of cancellous bone [17]. The contributions of canopy and endothelial cells should be assessed in future investigations.

It is interesting that in the present model the positive effects of ODN and ALN on bone formation result in increased trabecular thickness but not in prevention of OVX-induced loss of trabeculae, as also reported elsewhere [20]. Therefore, the observations reported here may concern surviving trabeculae, which have been found to correspond with the trabeculae submitted to strain [45]. One should thus be aware that the ODN effects reported in the present study might be conditioned by mechanical loading.

In conclusion, so far treatments of bone diseases have focused mainly on bone resorption itself or bone formation itself. However, bone diseases may not simply be due to a direct failure of resorption or formation but result from a lack of coordination between resorption and formation. Here, we propose that ODN is a bone resorption inhibitor which acts positively on the mechanism coordinating resorption with formation, through recruitment of osteoblast progenitors.
